# UV/Visible spectra of a series of natural and synthesised anthraquinones: experimental and quantum chemical approaches

**DOI:** 10.1186/2193-1801-3-233

**Published:** 2014-05-08

**Authors:** El Hassane Anouar, Che Puteh Osman, Jean-Frédéric F Weber, Nor Hadiani Ismail

**Affiliations:** Atta-ur-Rahman Institute for Natural Product Discovery, Universiti Teknologi MARA Kampus Puncak Alam, 42300 Bandar Puncak Alam, Selangor, D. E. Malaysia; Faculty of Applied Sciences, Universiti Teknologi MARA, 40450 Shah Alam, Selangor, D. E. Malaysia; Faculty of Pharmacy, Universiti Teknologi MARA, Puncak Alam Campus, 42300 Bandar Puncak Alam, Selangor, D. E. Malaysia

**Keywords:** Anthraquinones, UV/vis, TD-DFT, IEFPCM, Excited states

## Abstract

Root decoctions of anthraquinone-containing plants are often taken as postpartum tonic and aphrodisiac. Anthraquinones are known for their diverse biological activities, especially antioxidant and anticancer. A series of 35 anthraquinones was generated by isolation from Rubiaceae plants and synthesis. Their UV/vis spectrum depends on the nature and relative positions of auxochromic substituents on the basic skeleton. To predict the maximum absorption bands for the current series of anthraquinones, excited sate calculations were performed using TD-DFT, CIS, ZINDO methods. The results showed that the use of PBE0 or its combination with B3LYP and B3P86 hybrid functionals are the most suitable to reproduce accurately the experimental λ_MAX_. The structure–property relationships (SPRs) were established based on structural and electronic properties of the anthraquinones and showed (i) the importance of the number and position of OH groups and (ii) the positive contribution of the electrophilicity and hardness as electronic descriptors on position and amplitude of the maximum absorption bands.

## Introduction

Quinones are widely distributed in nature as pigments and intermediates in cellular respiration and photosynthesis (Koyama 2006; Koyama et al. 2008). Among them, anthraquinones form the largest group. They include 1,4- and 9,10-anthraquinones. The latter can be obtained in nature from various types of biological sources, including bacteria, marine sponges, fungi, lichens and higher plants from various families such as Rubiaceae, Gesneriaceae, Scrophulariaceae, Rhamnaceae, Polygonaceae and Leguminosae (Schripsema & Dagnino 1996; Thomson 1971). Anthraquinones attracted attention since the last century due to their applications in medicine and their presence as major constituents in many medicinal plants. They are known for their diverse biological activities especially antioxidant and anticancer properties (Ismail & Mohidin 2002; Osman et al. 2010; Ali et al. 2000). Anthraquinones-containing plants such as *Cassia alata*, *Prismatomeris sarmentosa*, *P. glabra*, *Morinda citrifolia*, *M. elliptica, Hedyotis capitellata*, and *Rennellia elliptica* are often consumed for health and enhancement vitality (Osman et al. 2010; Faridah Hanum & Hamzah 1999; Chan-Blanco et al. 2006; Azmi et al. 2011; Burkill 1966; Ahmad et al. 2005). Root decoctions are taken as post-partum tonic and aphrodisiac. In the textile industry, anthraquinones are used as dyes, providing various shades of colour depending on the nature and positions of auxochromic groups on their basic skeleton (Thomson 1971; Hunger 2003; Jacquemin et al. 2007a). To rationalize UV/vis spectral features, experimentalists analyse the chromophores present in their structures. UV/vis spectra of anthraquinones show four π → π* absorption bands in the wavelength range 220–350 nm (*i.e.*, due to S_π→π*_ electronic transitions) and one n → π* absorption band at longer wavelengths, close to 400 nm (*i.e.*, due to S_n→π*_ electronic transitions) (Diaz 1990).

The position and intensity of the absorption bands are strongly affected by the nature of the auxochromic substituents and environment (polar or apolar solvents). The UV/vis spectra of 1-hydroxyanthraquinones is determined primarily by their tautomeric or conformational structures (Fain et al. 2006). Intra- and intermolecular hydrogen bonding cause displacement towards longer wavelengths due the formation of a pseudo-ring through hydrogen bond, which increases the length of the conjugated system (Diaz 1990; 1991).

Quantum chemistry methods are viewed as efficient tools to reproduce experimental spectroscopic results. TD-DFT is considered as one of the most suitable methods to predict the UV/vis spectra of organic compounds for systems of intermediate size (Jacquemin et al. 2004; Jacquemin et al. 2007a; Anouar et al. 2012; Gierschner et al. 2007; Woodford 2005; Fabian 2001; Homem-de-Mello et al. 2005). Four previous studies by Jacquemin *et al.* discussed the choice of quantum chemistry methodology for reproducing the UV/visible spectra of different series of natural anthraquinones. They showed that a combination of PBE0 and B3LYP hybrid functionals in polarizable continuum model (PCM) could quite accurately reproduce the experimental results (Jacquemin et al. 2004; Jacquemin et al. 2007a; Jacquemin et al. 2007b; Perpète et al. 2006).

**Figure 1:**
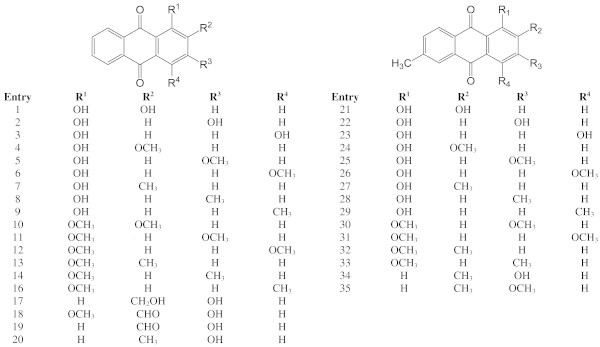
**Structures of natural and synthesised anthraquinones.**

A series of 35 anthraquinones had been isolated from natural sources and/or synthesized by our group (Figure 1). They all bear substituents of five different types, namely OH, OCH_3_, CH_3_, CH_2_OCH_3_, and CHO. They can be sorted into two groups. In the first group (**1**–**20**) only one aromatic ring is substituted, while the second group (**21**–**35**), includes compounds for which the second aromatic ring is additionally substituted by a 6-methyl substituent.

The present study aimed at (i) predicting the maximum absorption bands of the above mentioned anthraquinones, (ii) establishing the structure–property relationships (SPRs) between structural and electronic descriptors and the position of the maximum absorption bands, and (iii) obtaining the above data through classical computer-resources sparing methods combined with statistical treatment for increased accuracy. A correct of prediction of UV/Vis absorption would thus facilitate the dereplication of anthraquinones in drug discovery programs.

## Methodology

### Generation the anthraquinone library

*Natural anthraquinones:* 3-hydroxy-2-methylanthraquinone (**7**), 3-hydroxy-2-hydroxymethylanthraquinone (**17**), 2-formyl-3-hydroxy-1-methoxyanthraquinone (**18**), 2-formyl-3-hydroxyanthraquinone (**19**), 1-hydroxy-2-methoxy-6-methylanthraquinone (**24**), were isolated from *Rennellia elliptica* Korth. as described previously (Osman et al. 2010).

*Synthetic anthraquinones:* Twenty eight anthraquinones were synthetically prepared through Friedel Craft reaction and *O*-methylation using methods adapted from Singh (Singh 2005).

*i) Friedel Craft reactions:* Mixtures of phthalic anhydride (6.75 mmol) and benzene derivatives (0.01 mmol) were added to molten AlCl_3_: NaCl (2:1) at 150–180°C and stirred for 30 minutes. Upon the completion of the reaction, the deep red melts were carefully poured into 500 ml of HCl 3% and the mixtures were allowed to stand overnight to allow product precipitation. The precipitates were filtered and purified by MPLC.

*ii)* O*-methylation reactions:* Mixtures of hydroxyanthraquinones (1 mmol), methyl iodide (2 mmol) and potassium carbonate (1 mmol) in 30 mL acetone were refluxed for 8–120 hours and monitored by TLC. Upon reaction completion, the mixtures were dried under reduced pressure, redissolved in dichloromethane and extracted with distilled water to remove potassium carbonate residues. The organic layers were dried over anhydrous sodium sulfate and adsorbed onto silica prior to chromatographic purification.

### Experimental UV/visible spectra

Experimental UV/visible spectra were recorded on a Shimadzu UV-160A spectrometer in absolute ethanol.

### Theoretical calculations

The geometry optimization and frequency calculations of the ground states (GSs) of anthraquinone derivatives (AQs) were performed using semi-empirical (AM1), Hartree-Frock (HF) and density functional theory (DFT, with different hybrid functionals) methods. The minima of the optimized structures were confirmed by the absence of imaginary frequencies. The λ_MAX_ of the UV/vis spectra of anthraquinone derivatives were predicted within the vertical approximation. For a better description of absorption bands, one should consider vibronic coupling (*i.e.,* comparison between experimental and predicted vibrationally resolved spectra) to determine the shape vibrational modes. Jacquemin *et al.*, used a large panel of hybrid functionals (18 hybrid functional) and basis sets (7 basis sets) to predict the vibrationally resolved absorption spectra of a series of amino and hydroxyl anthraquinone dyes by using TD-DFT hybrid functionals, and found that the basis set has modest impact contrary to functional choice (Jacquemin et al. 2011; Jacquemin et al. 2012). However, the vibrational TD-DFT calculations are demanding high computing power and more CPU times. In the present study, a large panel of methods including ZINDO, CIS, HF and TD-DFT were tested to calculate the vertical electronic excitation energy (E_MAX_) and maximum wavelength (λ_MAX_). For the TD-DFT method, pure functionals (BLYP, BP86, PBE0), hybrid functionals (B3LYP, BHandLYP, B3P86, BHandP86, MPWBK, MPWB1K, B1LYP, PBE0) and long range corrected functionals (CAM-BLYP, CAM-B3LYP, WB97XD and LC-wPBE) were tested. Different basis sets were tested: (i) Double and triple-ζ Pople-type basis sets with or without polarized and diffuse functions [6-31G, 6-31G(d), 6-31G(d,p), 6-31 + G(d,p), 6-31++G(d,p), 6-311G(d,p), 6-311G(2d,2p), 6-311 + G(d,p), 6-311++G(d,p) and 6-311 + G(2d,3pd)]; (ii) Dunning’s basis set (cc-pVDZ) and (iii) Salahub’s basis sets [DGDZP, DGDZP2 and DGTZPV]. The solvent effects were taken into account using the Polarizable Continuum Model within IEF-PCM formalism (Tomasi et al. 2005). The PCM correctly models the major solvent effects as long as no specific solute-solvent interaction is considered. Recently, we tested IEF-PCM and SS-PCM specific state-PCM formalism to predict λ_MAX_ of terrein stereoisomers (Trabolsy et al. 2013). SS-PCM yielded better results than IEF-PCM by showing a more pronounced bathochromic shift form gas phase λ_MAX_. However, this formalism is strongly demanding of computing power. The results from the above listed methods were compared with the experimental data and three best methods were used to calculate the maximum absorption bands (λ_MAX_) of the 33 remaining anthraquinones. The solvent-anthraquinone interaction significantly affected the position of the absorption bands. The solvent can induce a bathochromic shift, especially in case of polar solvent (EtOH). In such a case, interactions between solvent and anthraquinone increase, leading to a better stabilised excited state and thus a bathochromic shift for the π → π* transition. The results were evaluated by simple and multiple linear regressions (SLR and MLR) and by plotting experimental λ_MAX_*vs* theoretical λ_MAX_.

All optimization, frequency and excited states calculations were performed using Gaussian 09 package (Frisch et al. 2009). The molecular orbitals of anthraquinones were visualized with Molden software (http://www.cmbi.ru.nl/molden/). The simple and multiple linear regression equations between the experimental and calculated maximum absorptions were obtained with the DataLab package (http://www.lohinger.com/datalab/en_home.html).

## Results and discussion

### Methodological approach

To determine the adequate methodology for predicting UV/vis spectra of this series of anthraquinone derivatives (Figure 1), E_MAX_ (λ_MAX_) were calculated for two prototypes 1,2-dihydroxyanthraquinone (**1**) and 1,2-dihydroxy-6-methylanthraquinone (**21**) using different methods and basis sets (Table 1). Only bands with the longest λ_MAX_ were compared. As can be seen from Table 1a and b, the HF, ZINDO and Long Range hybrid functionals failed to reproduce experimental E_MAX_ (λ_MAX_) for both anthraquinone prototypes **1** and **2**. Pure DFT functionals (with 0% HF exchange) underestimated E_MAX_. For DFT hybrid functionals (% Hartree-Fock exchange ≠ 0) the position of predicted E_MAX_ (λ_MAX_) depended on Hartree-Fock exchange contribution. For instance, E_MAX_ (λ_MAX_) values obtained for **1** using BLYP (0% HF), B3LYP (20% HF), B1LYP (25% HF) and BHandHLYP (50% HF) were 2.47 (502.62), 3.02 (411.08), 3.15 (394.07) and 3.78 eV (328.42 nm) respectively (Table 1a, PCM-Model column). Calculated E_MAX_ (λ_MAX_) that were the closest to the experimental ones were obtained with PBE0, B3LYP and B3P86 hybrid functionals, for which E_MAX_ (λ_MAX_) were 2.92 eV (424.52 nm), 3.02 eV (411.08 nm) and 3.03 eV (409.47 nm) respectively. These results fit with previous investigations of electronic excitation energies predictions of organic molecules (including anthraquinones derivatives) obtained by Jacquemin *et al*. (Jacquemin et al. 2004; Jacquemin et al. 2009; Jacquemin et al. 2010). The authors tested 29 functionals including M06 family functionals, and found that the functionals containing 22-25% exact exchange provide results closest to experimental data (Jacquemin et al. 2004; Jacquemin et al. 2009; Jacquemin et al. 2010). The above listed three best functionals were applied to calculate E_MAX_ (λ_MAX_) for the remaining 33 anthraquinones. To test basis set effects on calculated E_MAX_ (λ_MAX_), different basis sets were tested using B3P86 hybrid functional (Table 1b). The addition of polarization functions to 6-31G basis set induced an increase of E_MAX_ (decrease of λ_MAX_), while the addition of diffuse functions to polarized basis set 6-31G(d) and 6-31G(d,p) led to a decrease of E_MAX_ (increase of λ_MAX_). Adding more polarization and diffuse functions to double and triple basis sets had no significant effects. The basis set 6-31 + G(d,p) with diffuse and polarisation functions was chosen as the best compromise taking into account the accuracy of E_MAX_ (λ_MAX_) and computational time calculations. The low significance of diffuse and polarization in the estimating excitation energies was also previously mentioned in the theoretical investigation on substituted anthraquinones by Jacquemin *et al.* (Jacquemin et al. 2004).

**Table 1:** **Calculated λ**_**MAX**_ **(nm), E**_**MAX**_ **(eV),** ***f*** **and ET contributions obtained with different methods (a) and basis sets (b) for prototypes (1) and (21)**

(a)										
AQs	Methods	Gas phase				PCM-Model				λexp (Ee)
		λ_MAX_	Ee	f	ET	λ_MAX_	Ee	f	ET	
(1)	**TD-DFT**									433/2.87
	BLYP	471	2.63	0.09	H-1→L (69%)	503	2.47	0.12	H→L (66%)	
	B3LYP	438	2.83	0.10	H→L (69%)	411	3.02	0.16	H→L (70%)	
	B1LYP	370	3.35	0.13	H→L (69%)	394	3.15	0.17	H→L (70%)	
	BH and HLYP	311	3.99	0.18	H→L (69%)	328	3.78	0.22	H→L (69%)	
	BP86	469	2.64	0.09	H→L (69%)	501	2.48	0.12	H→L (66%)	
	B3P86	384	3.23	0.12	H→L (70%)	409	3.03	0.16	H→L (70%)	
	BH and HP86	380	3.26	0.15	H→L (68%)	404	3.07	0.21	H→L (69%)	
	MPWK	473	2.62	0.09	H-1→L (68%)	505	2.45	0.12	H→L (69%)	
	MPWB1K	455	2.72	0.08	H→L (69%)	344	3.60	0.21	H→L (69%)	
	PBE	469	2.64	0.09	H-1→L (69%)	501	2.48	0.12	H→L (69%)	
	PBE0	370	3.35	0.13	H→L (70%)	425	2.92	0.16	H→L (70%)	
	**Long-Range**									
	CAM-BLYP	286	4.33	0.21	H→L (64%)	298	4.16	0.27	H→L (65%)	
	CAM-B3LYP	326	3.81	0.17	H→L (68%)	344	3.61	0.22	H→L (69%)	
	CAM-wPBE	296	4.19	0.20	H→L (69%)	308	4.02	0.27	H→L (69%)	
	W97X	305	4.07	0.20	H→L (64%)	320	0.26	0.26	H→L (67%)	
	W97XD	325	3.82	0.18	H→L (67%)	342	3.62	0.23	H→L (70%)	
	**HF**	257	4.83	0.21	H→L (67%)	266	4.65	0.25	H→L (62%)	
	**CIS**	-	-	-	H→L (64%)	-	-	-	H→L (64%)	
	**ZINDO**									
	Opt (AM1)	346	3.58	0.25	H→L (64%)	-	-	-	H→L (65%)	
	Opt (PM3)	339	3.65	0.23	H→L (63%)	-	-	-	H→L (65%)	
	Opt (B3P86)	342	3.63	0.24	H→L (63%)	-	-	-	H→L+1 (49%)	
(21)	**TD-DFT**									431/2.88
	BLYP	465	2.66	0.09	H-1→L (69%)	495	2.50	0.12	H→L (69%)	
	B3LYP	432	2.87	0.10	H→L (69%)	406	3.05	0.16	H→L (70%)	
	B1LYP	367	3.38	0.14	H→L (69%)	390	3.18	0.17	H→L (70%)	
	BH and HLYP	309	4.01	0.18	H→L (68%)	326	3.80	0.22	H→L (69%)	
	BP86	463	2.68	0.09	H-1→L (69%)	493	2.51	0.12	H→L (69%)	
	B3P86	380	3.26	0.13	H→L (70%)	405	3.06	0.17	H→L (70%)	
	BH and HP86	378	3.28	0.16	H→L (68%)	401	3.09	0.21	H→L (69%)	
	MPWK	467	2.65	0.09	H-1→L (58%)	340	3.65	0.21	H→L (69%)	
	MPWB1K	398	3.11	0.14	H→L (68%)	341	3.63	0.21	H→L (69%)	
	PBE	463	2.68	0.09	H-1→L (69%)	493	2.51	0.13	H→L (69%)	
	PBE0	366	3.39	0.14	H→L (69%)	419	2.96	0.16	H→L (70%)	
	**Long-Range**									
	CAM-BLYP	285	4.35	0.22	H→L (64%)	297	4.18	0.28	H→L (65%)	
	CAM-B3LYP	324	3.83	0.18	H→L (68%)	341	3.63	0.23	H→L (69%)	
	CAM-wPBE	294	4.21	0.21	H→L (64%)	307	4.04	0.28	H→L (66%)	
	W97X	304	4.08	0.21	H→L (65%)	318	3.90	0.27	H→L (67%)	
	W97XD	323	3.84	0.19	H→L (69%)	340	3.65	0.24	H→L (68%)	
	**HF**	256	4.84	0.18	H→L (61%)	266	4.66	0.20	H→L (61%)	
	**CIS**	-	-	-	H→L+1 (65%)	-	-	-	H→L (65%)	
	**ZINDO**									
	Opt (AM1)	345	3.60	0.26	H→L (63%)	**-**	-	-	H→L (66%)	
	Opt (PM3)	322	3.85	0.15	H→L (55%)	**-**	-	-	H→L (66%)	
	Opt (B3P86)	341	3.64	0.25	H→L (67%)	**-**	-	-	H→L (66%)	
**(b)**										
(1)	6-31G	385	3.22	0.13	H→L (69%)	408	3.04	0.17	H→L (70%)	433/2.87
	6-31G(d)	380	3.26	0.12	H→L (69%)	403	3.08	0.16	H→L (70%)	
	6-31G(d, p)	380	3.26	0.12	H→L (69%)	403	3.08	0.16	H→L (70%)	
	6-31 + G(d, p)	384	3.23	0.12	H→L (69%)	409	3.03	0.16	H→L (69%)	
	6-31++G(d, p)	384	3.23	0.12	H→L (69%)	409	3.03	0.16	H→L (66%)	
	6-311G(d, p)	379	3.27	0.12	H→L (69%)	403	3.08	0.16	H→L (70%)	
	6-311G(2d, 2p)	379	3.27	0.11	H→L (68%)	402	3.08	0.15	H→L (70%)	
	6-311 + G(d, p)	382	3.25	0.12	H→L (69%)	406	3.05	0.16	H→L (70%)	
	6-311++G(d, p)	382	3.25	0.12	H→L (69%)	406	3.05	0.16	H→L (70%)	
	6-311+G(2d, 3pd)	382	3.25	0.11	H→L (69%)	406	3.05	0.15	H→L (70%)	
	cc-pVTZ	379	3.27	0.11	H→L (69%)	403	3.08	0.15	H→L (70%)	
	aug-cc-pVTZ	-	-	-	H→L (68%)	-	-	-	H→L (68%)	
	DGDZPV	382	3.24	0.12	H→L (69%)	407	3.05	0.16	H→L (70%)	
	DGDZPV2	383	3.24	0.12	H→L (69%)	408	3.04	0.16	H→L (70%)	
	DGTZVP	382	3.24	0.12	H→L (69%)	407	3.05	0.16	H→L (70%)	
(21)	6-31G	381	3.25	0.14	H→L (69%)	403	3.07	0.18	H→L (70%)	431/2.88
	6-31G(d)	376	3.29	0.13	H→L (69%)	398	3.11	0.17	H→L (70%)	
	6-31G(d, p)	376	3.29	0.13	H→L (69%)	399	3.11	0.17	H→L (70%)	
	6-31+ G(d, p)	380	3.26	0.13	H→L (69%)	405	3.06	0.17	H→L (70%)	
	6-31++G(d, p)	380	3.26	0.13	H→L (69%)	405	3.06	0.17	H→L (70%)	
	6-311G(d, p)	375	3.30	0.12	H→L (69%)	398	3.11	0.16	H→L (70%)	
	6-311G(2d, 2p)	375	3.30	0.12	H→L (68%)	397	3.12	0.15	H→L (70%)	
	6-311+G(d, p)	378	3.28	0.13	H→L (69%)	402	3.09	0.16	H→L (70%)	
	6-311++G(d, p)	378	3.28	0.13	H→L (69%)	402	3.09	0.16	H→L (70%)	
	6-311+G(2d, 3pd)	378	3.28	0.12	H→L (69%)	-	-	-	H→L (69%)	
	cc-pVTZ	376	3.30	0.12	H→L (69%)	-	-	-	H→L (70%)	
	aug-cc-pVTZ	-	-	-	H→L (69%)	-	-	-	H→L (65%)	
	DGDZPV	378	3.28	0.13	H→L (69%)	402	3.09	0.16	H→L (70%)	
	DGDZPV2	379	3.27	0.13	H→L (69%)	403	3.08	0.16	H→L (70%)	
	DGTZVP	382	3.25	0.13	H→L (69%)	402	3.08	0.16	H→L (70%)	

**Figure 2:**
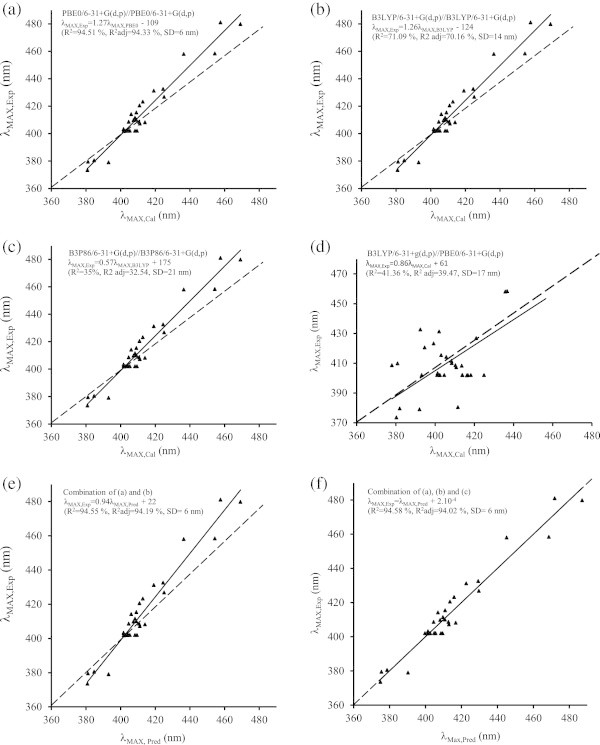
**Correlation curves obtained with PBE0, B3LYP, B3P86 hybrid functionals (a-d) and its combinations (e-f).**

The above methodology was applied to the 33 remaining anthraquinones in gas phase as well as with PCM (methanol) in four different series of calculations. For three of them, the functional used for structure optimisation and E_MAX_ (λ_MAX_) calculations was identical, *i.e.* at B3LYP/6-31 + G (d, p), B3P86/6-31 + G (d, p), and PBE0/6-31 + G (d, p) levels. The fourth was calculated at B3LYP/6-31 + G (d, p)//PBE0/6-31 + G (d, p) level as the B3LYP/6-31 + G (d, p) functional is supposed to provide good performance. Table 2 gathers results obtained using the latter method. Simple linear regression was applied for each of the three data sets obtained with the above methodology; the corresponding linear regression curves are shown in Figure 2a-2c. The correlation obtained with B3LYP (R^2^ = 71.09%, R^2^_adj_ = 70.16%, SD = 14 nm) is not very good (Figure 2b) compared to PBE0 (Figure 2a), probably due to the high Hartee-Fock exchange percentage in PBE (25% HF) compared to B3LYP (20% HF) (Jacquemin et al. 2007a). The correlation obtained with B3P86 (R^2^ = 35%, R^2^_adj_ = 32.54%, SD = 21 nm) is even worse (Figure 2c). When optimized structures at B3LYP and excited state calculation at PBE0 levels are used, calculated and experimental λ_MAX_ weakly correlated (R^2^ = 41.36%, R^2^_adj_ = 39.47, SD = 17 nm) (Figure 2d). The best correlation (R^2^ = 94.51%, R^2^_adj_ = 94.33%, SD = 6 nm) was obtained with PBE0 hybrid functional (Figure 2a) taking into account the solvent effect, thus corroborating Jacquemin’s group results (Jacquemin et al. 2007a).

**Table 2:** **Calculated vs experimental λ**_**MAX**_ **(nm) for the 35 anthraquinones obtained at the PBE0/6-311 + G(d,p)//PBE0/6-311 + G(d,p) level**

Compounds	Gas				PCM (methanol)				λ_EXP_
	λ_MAX_	Ee	f	E.T	λ_MAX_	Ee	f	E.T	
(1)	419	2.96	0.11	H→L (70%)	425	2.92	0.16	H→L (70%)	433
(2)	400	3.10	0.11	H→L (68%)	406	3.05	0.13	H→L (69%)	414
(3)	453	2.74	0.19	H→L (70%)	458	2.71	0.24	H→L (70%)	481
(4)	427	2.91	0.13	H→L (69%)	413	3.00	0.19	H→L (69%)	423
(5)	402	3.08	0.11	H→L (68%)	411	3.02	0.13	H→L (69%)	407
(6)	442	2.81	0.18	H→L (70%)	454	2.84	0.23	H→L (70%)	458
(7)	401	3.10	0.15	H→L (70%)	408	3.04	0.21	H→L (70%)	410
(8)	396	3.13	0.14	H→L (70%)	402	3.09	0.18	H→L (70%)	403
(9)	406	3.06	0.16	H→L (70%)	411	3.02	0.21	H→L (70%)	409
(10)	397	3.13	0.16	H→L (70%)	408	3.04	0.04	H→L (46%)	402
(11)	422	2.94	0.00	H-1→L (61%)	404	3.07	0.02	H-2→L (48%)	402
(12)	428	2.90	0.01	H-1→L (50%)	425	2.92	0.00	H-1→L (50%)	427
(13)	406	3.05	0.00	H-1→L (65%)	403	3.08	0.02	H-2→L (44%)	402
(14)	424	2.93	0.00	H→L (50%)	402	3.08	0.01	H-2→L (54%)	402
(15)	425	2.92	0.00	H-1→L (60%)	409	3.03	0.02	H-1→L (58%)	402
(16)	400	3.10	0.11	H→L (68%)	408	3.04	0.14	H→L (68%)	412
(17)	362	3.43	0.02	H→L (68%)	381	3.26	0.03	H→L (69%)	374
(18)	387	3.20	0.05	H→L (58%)	385	3.22	0.05	H→L (63%)	381
(19)	372	3.34	0.03	H→L (69%)	381	3.25	0.04	H→L (69%)	380
(20)	372	3.33	0.02	H→L (68%)	393	3.15	0.03	H→L (69%)	379
(21)	414	2.99	0.11	H→L (70%)	419	2.96	0.16	H→L (70%)	431
(22)	395	3.14	0.09	H→L (68%)	409	3.03	0.15	H→L (69%)	415
(23)	450	2.76	0.20	H→L (70%)	469	2.64	0.25	H→L (70%)	480
(24)	371	3.34	0.16	H→L (70%)	411	3.02	0.21	H→L (69%)	421
(25)	403	3.07	0.11	H→L (68%)	414	2.99	0.13	H→L (68%)	408
(26)	432	2.87	0.16	H→L (69%)	436	2.84	0.21	H→L (70%)	458
(27)	398	3.12	0.17	H→L (70%)	405	3.06	0.23	H→L (70%)	409
(28)	395	3.14	0.15	H→L (69%)	402	3.09	0.20	H→L (69%)	402
(29)	404	3.07	0.17	H→L (70%)	409	3.03	0.23	H→L (70%)	410
(30)	422	2.94	0.00	H-1→L (60%)	404	3.07	0.02	H-3→L (47%)	402
(31)	-	-	-	H→L (56%)	-	-	-	H-1→L (53%)	424
(32)	407	3.05	0.00	H-3→L (61%)	403	3.08	0.02	H-2→L (51%)	402
(33)	407	3.05	0.00	H-2→L (53%)	402	3.08	0.01	H-2→L (46%)	402
(34)	374	3.32	0.02	H→L (58%)	395	3.14	0.00	H-2→L (67%)	402
(35)	382	3.2463	0.03	H→L (69%)	405	3.06	0.04	H→L (69%)	402

In an attempt to improve the correlations, we combined the results obtained with PBE0, B3LYP and B3P86 hybrid functionals and subjected them to multiple linear regressions. The following equations 1–4 were obtained:

(eq. 1: R^2^ = 72.71%; R^2^_adj_ = 70.89%; SD = 13 nm)

(eq. 2: R^2^ = 94.58%; R^2^_adj_ = 94.02%; SD = 6 nm)

(eq. 3: R^2^ = 94.55%; R^2^_adj_ = 94.19%; SD = 6 nm)

(eq. 4: R^2^ = 94.53%; R^2^_adj_ = 94.16%; SD = 6 nm)

The combination of B3LYP with B3P86 led to a standard deviation of 13 nm (eq. 1), thus less accurate than PBE0 alone. The combination of PBE0 with B3LYP and/or B3P86 hybrid functionals (eq. 2–4) yielded similar standard deviations of 6 nm (Figure 2e-2f), comparable to those obtained from PBE0 alone. The high contribution of PBE0 hybrid functional in these equations should be noted. This comes in contrast to Jacquemin’s group results whereby they improved the SD from 12 nm as best result with a single hybrid functional to 6 nm by combining two of them (Jacquemin et al. 2004; Jacquemin et al. 2007b; Perpète et al. 2006). It seems that an SD of 6 nm is the best that can be achieved with current tools, i.e., hybrid functionals and basis sets.

### Structure–property relationships on absorption band λ_MAX_

**Figure 3:**
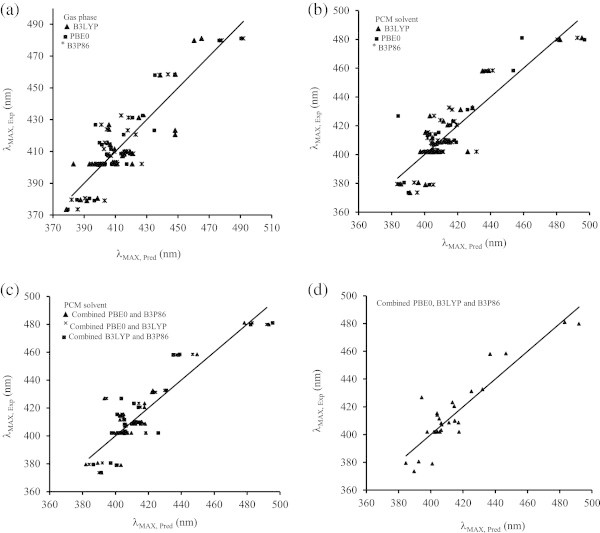
**Correlation curves obtained based on electronic descriptors with PBE0, B3LYP and B3P86 hybrid functionals: (a) In gas phase, (b) in polarizable continuum model, (c) with the combination of two functionals, and (d) the combination of the three functionals.**

It is well established that increasing the number of aromatic OH substituents of organic compounds induces a bathochromic shift (red shift) of the λ_MAX_, while methylation of OH groups induces an hypsochromic shift (Anouar et al. 2012). Anthraquinone **1** with two aromatic OH groups has a λ_MAX_ at 432 nm, while anthraquinone **7** with one aromatic OH groups shows a λ_MAX_ at 410 nm. The methylation of **1** leads to a hypsochromic shift of λ_MAX_ by 30 nm [1,2-dimethoxanthraquinone (**10**)]. These calculated results are in good agreement with the experimental ones. The bathochromic shift of the λ_MAX_ is probably due to the mesomeric effect (+M) of OH groups which extends the delocalization of frontier orbitals of the HOMO and LUMO. The absorption band λ_MAX_ in anthraquinone derivatives is significantly influenced by the relative position of OH groups to each other (*ortho*, *meta* and *para*). For instance, in one hand the experimental results show a bathochromic shift form the absorption of *ortho*-dihydroxy anthraquinones (e.g. **1**) of their *para*-substituted isomers (e.g. **3**); on the other hand, two OH groups in *meta* positions (e.g. **2**) induce a hypsochromic shift with respect to the *ortho* substitution (e.g. **1**). The decrease and increase of λ_MAX_ can be explained by the mesomeric effect (+M) of hydroxyl groups. For instance, 1,2-dihydroxyanthraquinone (**1**) has an absorption band at 433 nm (Table 2), whereas 1,3-dihydroxyanthraquinone (**2**) shows a maximum absorption at 414 nm. In case of 1,4-dihydroxyanthraquinone (**3**) with two *para* OH groups, a significant bathochromic shift of λ_MAX_ (481 nm) happens due to the extension of the molecular orbital delocalization. The calculated effects of the OH group positions are in good agreement with experimental ones. Similar remarks can be made for compounds with 6-methyl group (**21**–**23**). The mesomeric effect of the methyl group at C6 is negligible (*e.g.*, compare λ_MAX_ for **1**–**3** with that of **21**–**23**). The electrostatic interactions between a polar solvent (ethanol) and the anthraquinone lead to stabilise the excited state of the anthraquinone which makes the electron transfer from the ground sate to the excited state faster, and thus to a bathochromic shift (red shift) of the maximum absorption bands (λ_MAX_). For instance, the maximum absorption bands for 1,2-dihydroxyanthraquinone (**1**) in gas and solvent are 419 and 425 nm respectively. The polar solvent has an hyperchromic effect on the absorption band (increase of the oscillator strength *f*). Taking again example of **1**, the oscillator strength increases in the presence of solvent (0.16) compared to gas phase (0.10). To correlate the electronic descriptors with the position of λ_MAX_ of the current series of anthraquinones, we calculated the ionisation potential (IP), electron affinity (EA), hardness (η), electrophilicity (ω), polarizability (α), electronegativity (χ) and dipole moment (μ) for anthraquinones derivatives in gas and solvent using PBE0 (Table 3), B3LYP and B3P86 hybrid functionals. The simple linear regression curves and their respective equations obtained with each descriptor separately using the above hybrid functionals were calculated. The SLR showed that ionization potential (IP), electron affinity (EA), hardness (η) and electrophilicity (ω) have higher contribution (positive or negative) than polarizability (α), electronegativity (χ), and dipole moment (μ). The highest contribution was found from the hardness parameter (η) with a correlation of 72%. In accordance with these results, Fayet *et al.* found that among eight descriptor tested on a series of 24 anthraquinones, the chemical hardness (η) provided the largest R^2^ of 92% (Fayet et al. 2010). As the R^2^ of the above SLR were not satisfactory, multiple linear regressions (MLR) were applied by combining the descriptors contributions from each functional separately (Eq. 5–7 and Figure 3) and led to some improvements. The lowest standard deviation was obtained with Eq. 5. These results are also in accordance with MLR analysis results obtained by Fayet *et al.* who obtained a SD of 14.2 nm by combining hardness (η) and polarizability (α) (Fayet et al. 2010). In our case, the MLR of Eq. 5 provides a SD of 12 nm, while a SD of 13.7 was obtained by considering only hardness (η) and polarizability (α) parameters. Eventually, MLR was applied to the three above functionals, leading to Eq. 8 below.

**Table 3:** **Electronic descriptors obtained at PBE0/6-31 + G (d, p) level**

Gas	IP (eV)	EA (eV)	χ (eV)	η (eV)	ω (eV)	α	μ (Debye)	λ_MAX, Exp_ (nm)
(1)	6.77	3.17	4.97	3.60	3.43	183.17	2.13	432.60
(2)	7.02	3.17	5.09	3.85	3.37	182.27	2.55	414.20
(3)	6.55	3.31	4.93	3.25	3.75	185.91	2.05	481.00
(4)	6.68	3.13	4.90	3.55	3.39	198.49	1.82	423.20
(5)	6.94	3.11	5.03	3.83	3.30	197.08	2.46	407.20
(6)	6.39	3.01	4.70	3.38	3.26	198.49	1.67	458.40
(7)	6.86	3.11	4.98	3.75	3.31	190.73	0.66	409.80
(8)	6.92	3.10	5.01	3.82	3.28	190.34	1.02	403.20
(9)	6.80	3.11	4.96	3.69	3.33	189.16	1.41	408.60
(10)	6.38	2.60	4.49	3.78	2.66	210.42	3.93	402.00
(11)	7.02	2.89	4.95	4.12	2.97	206.29	1.79	402.00
(12)	7.13	2.86	5.00	4.26	2.93	202.44	1.14	426.80
(13)	6.86	2.83	4.84	4.03	2.91	200.96	1.99	402.00
(14)	7.22	2.89	5.05	4.32	2.96	199.50	1.02	402.00
(15)	7.19	2.85	5.02	4.33	2.91	197.71	0.61	402.00
(16)	7.04	3.20	5.12	3.83	3.42	213.63	3.02	411.50
(17)	7.31	3.04	5.18	4.26	3.14	192.78	2.91	373.50
(18)	7.18	3.24	5.21	3.94	3.45	213.05	4.51	380.50
(19)	7.42	3.34	5.38	4.08	3.55	195.10	3.08	379.50
(20)	7.03	2.89	4.96	4.14	2.97	188.90	1.16	379.00
(21)	6.70	3.07	4.89	3.63	3.29	199.30	2.69	431.20
(22)	6.95	3.07	5.01	3.88	3.23	197.92	2.86	415.40
(23)	6.49	3.22	4.85	3.27	3.60	201.92	2.83	479.80
(24)	6.60	2.59	4.60	4.01	2.64	210.41	3.29	420.50
(25)	6.86	3.02	4.94	3.84	3.18	212.83	3.16	408.20
(26)	6.49	3.01	4.75	3.47	3.25	212.43	3.11	458.00
(27)	6.79	3.01	4.90	3.78	3.18	206.83	1.41	408.60
(28)	6.85	3.00	4.93	3.85	3.16	206.25	1.82	402.00
(29)	6.73	3.02	4.88	3.71	3.20	205.01	2.21	410.00
(30)	6.95	2.83	4.89	4.12	2.90	221.83	2.04	402.00
(31)	-	-	-	-	-	-	-	-
(32)	6.81	2.76	4.78	4.04	2.83	216.79	1.93	402.00
(33)	6.86	2.77	4.82	4.09	2.84	216.89	1.58	401.80
(34)	6.96	2.80	4.88	4.16	2.86	204.46	1.47	402.00
(35)	6.81	2.75	4.78	4.06	2.81	218.69	1.86	402.00
**PCM solvent**								
(1)	6.80	3.21	5.00	3.59	3.49	258.37	2.81	432.60
(2)	7.05	3.22	5.14	3.83	3.44	256.07	3.39	414.20
(3)	6.61	3.33	4.97	3.28	3.76	264.29	2.76	481.00
(4)	6.93	3.24	5.09	3.69	3.51	273.00	2.65	423.20
(5)	7.03	3.22	5.12	3.81	3.45	274.50	3.35	407.20
(6)	6.50	3.16	4.83	3.34	3.49	279.01	2.04	458.40
(7)	6.94	3.20	5.07	3.74	3.44	266.69	0.90	409.80
(8)	7.02	3.20	5.11	3.82	3.41	265.47	1.38	403.20
(9)	6.88	3.18	5.03	3.70	3.42	265.46	1.81	408.60
(10)	6.95	3.04	5.00	3.92	3.19	284.85	2.16	402.00
(11)	7.02	3.04	5.03	3.98	3.17	286.15	2.85	402.00
(12)	7.29	3.01	5.15	4.28	3.10	276.73	1.67	426.80
(13)	6.96	2.99	4.98	3.97	3.12	278.70	2.85	402.00
(14)	7.05	3.00	5.03	4.05	3.12	277.72	2.30	402.00
(15)	6.91	2.96	4.94	3.95	3.09	277.37	2.51	402.00
(16)	7.05	3.23	5.14	3.82	3.47	295.06	4.01	411.50
(17)	7.21	3.12	5.17	4.10	3.26	266.43	3.81	373.50
(18)	7.29	3.31	5.30	3.99	3.52	293.49	4.78	380.50
(19)	7.36	3.33	5.35	4.04	3.54	270.66	3.95	379.50
(20)	7.02	3.05	5.04	3.98	3.19	262.92	1.67	379.00
(21)	6.77	3.15	4.96	3.62	3.40	279.06	3.41	431.20
(22)	6.97	3.14	5.06	3.83	3.34	276.34	1.21	415.40
(23)	6.39	3.40	4.90	2.99	4.01	292.56	3.75	479.80
(24)	6.91	3.20	5.06	3.71	3.45	293.84	3.38	420.50
(25)	6.98	3.17	5.07	3.82	3.37	294.49	4.28	408.20
(26)	6.63	3.13	4.88	3.50	3.40	295.91	4.26	458.00
(27)	6.91	3.15	5.03	3.77	3.36	287.22	1.82	408.60
(28)	6.98	3.14	5.06	3.84	3.33	285.90	2.39	402.00
(29)	6.85	3.12	4.98	3.72	3.34	286.05	2.84	410.00
(30)	6.98	3.00	4.99	3.97	3.13	305.69	3.44	402.00
(31)	-	-	-	-	-	-	-	-
(32)	6.95	2.96	4.95	3.99	3.08	298.58	2.83	402.00
(33)	7.03	2.97	5.00	4.06	3.08	297.63	2.46	401.80
(34)	6.98	2.99	4.99	3.99	3.12	282.64	2.08	402.00
(35)	6.87	2.98	4.92	3.89	3.12	300.61	2.31	402.00

(eq. 5: R^2^ = 82.41%; R^2^_adj_ = 77.68%; SD = 12 nm)

(eq. 6: R^2^ = 73.60%; R^2^_adj_ = 66.76%; SD = 14 nm)

(eq. 7: R^2^ = 77.56%; R^2^_adj_ = 71.52%; SD = 13 nm)

(eq. 8: R^2^ = 83.95%; R^2^_adj_ = 82.23%; SD = 11 nm)

The combination of the three hybrid functionals yields a better correlation than each functional separately. The λ_MAX_ is positively influenced (red shift) by the hardness (η) and electrophilicity (ω) descriptors, and negatively influenced (blue shift) by the ionization potential (IP), electron affinity (EA) and electronegativity (χ). The regression equations show that the polarizability (α) and dipole moment (μ) contributions are not significant. Equation 8 showed a higher contribution of PBE0 compared to B3LYP and B3P86 hybrid functionals.

## Conclusion

In the present study, we showed that the hybrid functional PBE0 was able to reproduce the absorption band λ_MAX_ with a standard deviation of 6 nm. This value could be matched but not improved using various combinations of hybrid functionals PBE0, B3LYP and B3P86. It is also very close the experimental error, which would typically be of few nm. The structure–property relationships study based on structural and electronic descriptors analysis showed that the bathochromic or hypsochromic shifts are influenced by the number and position of OH groups, the hardness, electrophilicity, ionization potential, electron affinity and electronegativity descriptors.
